# Infectious Risk Assessment of Unsafe Handling Practices and Management of Clinical Solid Waste

**DOI:** 10.3390/ijerph10020556

**Published:** 2013-01-31

**Authors:** Md. Sohrab Hossain, Nik Norulaini Nik Ab Rahman, Venugopal Balakrishnan, Vignesh R. Puvanesuaran, Md. Zaidul Islam Sarker, Mohd Omar Ab Kadir

**Affiliations:** 1 Department of Environmental Technology, School of Industrial Technology, Universiti Sains Malaysia, Penang 11800, Malaysia; E-Mail: msh.id09@student.usm.my; 2 School of Distance Education, Universiti Sains Malaysia, Penang 11800, Malaysia; E-Mail: norulain@usm.my; 3 Institute for Research in Molecular Medicine, Universiti Sains Malaysia, Penang 11800, Malaysia; E-Mails: venugopal@usm.my (V.B.); vignesh.r.puvanesuaran@gmail.com (V.R.P.); 4 Department of Pharmaceutical Technology, Faculty of Pharmacy, International Islamic University Malaysia, Kuantan Campus, Bandar Indera Mahkota, Kuantan 25200, Pahang, Malaysia; E-Mail: zaidul@iium.edu.my

**Keywords:** clinical solid waste, clinical solid waste management, healthcare waste, pathogenic bacteria, sharp waste

## Abstract

The present study was undertaken to determine the bacterial agents present in various clinical solid wastes, general waste and clinical sharp waste. The waste was collected from different wards/units in a healthcare facility in Penang Island, Malaysia. The presence of bacterial agents in clinical and general waste was determined using the conventional bacteria identification methods. Several pathogenic bacteria including opportunistic bacterial agent such as *Pseudomonas aeruginosa*, *Salmonella* spp., *Klebsiella pneumoniae*, *Serratia marcescens*, *Acinetobacter baumannii*, *Staphylococcus aureus*, *Staphylococcus epidermidis*, *Enterococcus faecalis*, *Streptococcus pyogenes* were detected in clinical solid wastes. The presence of specific pathogenic bacterial strains in clinical sharp waste was determined using 16s rDNA analysis. In this study, several nosocomial pathogenic bacteria strains of *Escherichia coli*, *Klebsiella pneumoniae*, *Proteus mirabilis*, *Lysinibacillus sphaericus*, *Serratia marcescens*, and *Staphylococcus aureus* were detected in clinical sharp waste. The present study suggests that waste generated from healthcare facilities should be sterilized at the point of generation in order to eliminate nosocomial infections from the general waste or either of the clinical wastes.

## 1. Introduction

There is growing worldwide awareness about effective control and safe handling of clinical solid waste due to the common concern for hospital hygiene [1,2]. The clinical solid waste defined by the Medical Waste Tracking Act of 1988 are solid waste materials, which are generated during diagnosis, treatment, vaccination, research or in the production or testing of biological products for humans and animals. The term clinical solid waste includes syringes, live vaccines, blood and other waste contaminated with bodily fluids, culture dishes, sharp objects, discarded surgical gloves, discarded surgical instruments, cultures, stocks, swabs used to inoculate cultures, removed body organs, *etc.* The collection, segregation and disposal of clinical solid waste entails labor intensive operations, involving many possibilities of direct contact with the waste increasing the risk of infections to the waste handlers [2,3,4].

Earlier studies indicated that the clinical solid waste management at healthcare facilities is inadequate in developing countries [5,6,7,8,9]. In many developing countries, the clinical waste is handled and disposed together with non-clinical waste [1,6,9], which is creating a vital and even fatal health risk to health care workers and the general public [9,10,11,12]. Healthcare workers are prevalently exposed to diseases like cholera, plague, tuberculosis, hepatitis, skin infections, diphtheria, food poisoning, *etc.*, in either epidemic or even endemic form [9,12]. Surveys reveal that the incidences of contracting diseases are most prevalent among the waste handlers compared to other hospital staff [9,10]. Waste handler’s general exposure due to their occupational job functions could result in an infection during waste handling through punctures, cuts, inhalation or dermal contact than other healthcare staff. However, the literature on the spread of infectious diseases to clinical waste workers is extremely limited. Nevertheless, studies have summarized that poor management practices and improper precautions taken by clinical waste workers during waste collection, segregation and disposal might be the main reason of the spread of infectious diseases among clinical waste handlers [2,6,9]. 

Clinical solid waste is perceived by many as hazardous or infectious, and requires steps to minimize occupational incidents and environmental contamination [13,14,15,16]. Numerous studies have been conducted worldwide to define the best appropriate clinical waste management practice in order to minimize the health hazards and associate environmental contamination [1,5,6,7,8,9]. However, studies reported that one of the main reasons on the mismanagement of clinical solid waste is the lack of awareness of the waste handlers regarding the infectious risk of clinical solid waste [2,5,9,16]. 

The infectious risk posed by clinical solid waste to human health and the environment, which needs to be assessed, is the potential presence of pathogenic microorganisms. Clinical solid waste might contain a great variety of pathogenic microorganisms [16,17,18,19]. Among the various types of clinical waste, the handling of and disposal of sharps clinical waste is of great concern in developing and transitional countries of the World. Sharps waste such as hypodermic needles is considered a highly hazardous waste resulting from the common contamination from the patients’ blood [18]. Clinical sharps waste not only causes cuts and punctures but also infects wounds with the pathogens in the contaminated waste. Therefore, clinical sharp waste is considered as a waste with double or higher risk to healthcare workers. 

The potential microbiological risks associated with the clinical solid waste are still limited to the healthcare worker and other clinical personnel due to availability of inadequate data on type and quantity of pathogenic microorganism present in the clinical solid waste. Studies have been documented as regards to the infectious risks in clinical waste management [16,17,19], unfortunately scientifically substantiated evidence of the actual content of microorganisms, survival of microorganisms in clinical solid waste and the infectious risks to healthcare workers and the public are rare. Identifying the types and quantity of microorganisms present in clinical solid waste in order to achieve a reliable human health risk assessment are essential. The focus of this work was to identify and categorize the presence of pathogenic bacteria in clinical solid waste collected from various department/wards in a healthcare facility of Penang Island, Malaysia. The identification of bacteria in clinical sharp waste was conducted via molecular means, using 16s rDNA sequencing to evaluate the pathogenicity with the detection of the specific pathogenic bacterial strains. The outcome of this study may be useful to determine the reliable technology for the safe handling, disposal and the possible infectious threat of clinical solid waste.

## 2. Experimental Section

### 2.1. Clinical Solid Waste Collection and Preparation

The clinical solid waste was collected from one of the largest healthcare facilities in Penang Island, Malaysia. Waste collection was conducted for a period of one month in April 2012, in cooperation with the hospital administration. Clinical solid waste sample was collected from different wards/units of the hospital: Dental ward, Microbiological lab, ICU unit, Dermatology unit, Isolation ward, Obstetrical & Gynecology wards. The variations of the microbiological bacterial agents’ presence in clinical solid waste generated in the units/wards were investigated. The presence of bacterial agents in general waste which was collected from the central storage room was also determined. The materials used during collection and transportation of the waste from the hospital according to the healthcare waste management guideline and legislation of Ministry of Health, Malaysia [20]. 

The collected clinical waste samples were transferred to a Biohazard clinical waste bin. One liter of sterilized distilled water was then added to every kilogram of clinical waste sample and mixed vigorously using a glass rod. The prepared sample was stored at room temperature (25 ± 1 °C) for 1 h.

### 2.2. Identification of the Bacteria in Clinical Solid Waste Using the Conventional Method

**Figure 1:**
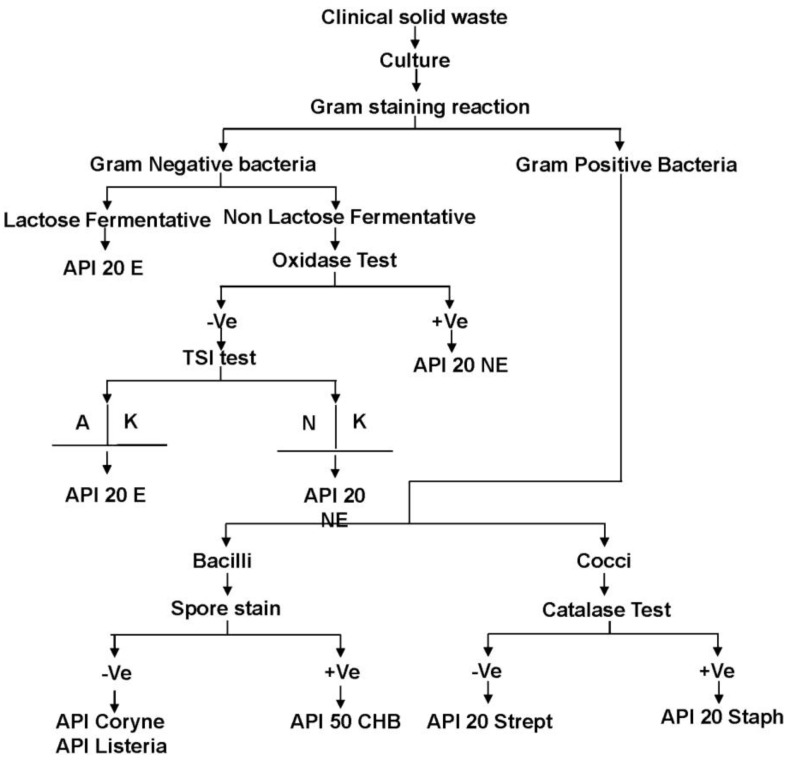
Flow chart for the identification of bacteria in clinical solid waste. +Ve—positive; −Ve—Negative; A—Acid growth; K—Alkaline growth; N—Neutral.

Bacterial identification was conducted following a manual of microbiological analysis [21]. A 0.1 mL aliquot of prepared contaminated sample was taken from the respective clinical solid waste storage bin to be cultured on agar media. Blood and MacConkey agar medium was used to culture the bacteria for Gram positive and Gram negative bacteria, respectively. Bacteria cultures on agar media were prepared in triplicate. Single isolated bacterium colonies were obtained following several steps of subculturing. Gram stain reaction and other biochemical tests including catalase test, oxidase test, triple sugar iron test were used for the morphological analysis of bacteria. Bacterial species were confirmed using selective Analytab Products Inc. (API) kit analysis. The details of bacterial identification are presented in Figure 1. 

### 2.3. Identification of the Bacteria in Clinical Sharp Waste Using Genomic DNA Profiling

The collection of clinical sharp waste was conducted in cooperation with the studied hospital nurses in the month of May, 2012. Nurses on duty during the period were supplied with a sharp waste bin. Contaminated clinical sharp waste were collected from ICU unit, Dermatology unit, Isolation ward and Obstetrical & Gynecology ward and put into the bin. Later, sterilized distilled water in the ratio of 1:2 L·kg^−1^ was added into the collected clinical sharp waste bin and mixed vigorously using a glass rod. The sample was stored at room temperature (25 ± 1 °C) for 1 h prior to culture the bacteria on agar plates. A 0.1 mL sample of contaminated waste was taken from the respective clinical solid waste storage bin to culture on agar media. Blood and MacConkey agar medium was used to culture the bacteria for Gram positive and Gram negative bacteria, respectively. However, the culture of the contaminated waste was carried out thrice on blood and MacConkey agar media. Moreover, a few steps of subculturing were conducted in order to obtain single isolated bacterium colonies.

In order to perform 16s rDNA analysis of the bacteria, the single isolated bacterium colony was transferred into nutrient broth and incubated for 18 h with agitation of 200 rpm and appropriate aeration. Later, the culture was pelleted through centrifugation at 1,500 × *g* for 20 min at 4 °C before the broth was discarded. QIAamp DNA Mini Kit (Qiagen, Hilden, Germany) was then utilized to extract the bacterial genomic DNA according to the manufacturer’s instructions. The extracted genomic DNA was then tested for integrity by quantifying its absorbance at A_260_/A_280_ using a NanoPhotometer (Implen, Munich, Germany). The 16s rDNA sequencing was carried out by Macrogen (Seoul, Korea). The obtained sequences were than analyzed using the standard nucleotide BLAST tool (NCBI). The identities of the bacteria were determined by observing their similarity with the posted sequences (max identity; %).

## 3. Results and Discussion

Clinical solid waste may contain varieties of bacteria. The types and quantity of bacteria depend on the clinical solid waste compositions and its generation source. Table 1 shows the types of bacteria recovered from the different wards/units in the Hospital studied. Nine types of Gram negative and seven types of Gram positive bacteria were detected in the clinical solid waste. The gram negative bacteria detected were *Escherichia coli* (*E. coli*), *Proteus mirabilis* (*P. mirabilis*), *Pseudomonas aeruginosa* (*P. aeruginosa*), *Salmonella* spp., *Klebsiella pneumoniae* (*K. pneumoniae*), *Serratia liquefaciens* (*S. liquefaciens*), *Serratia marcescens* (*S. marcescens*), *Acinetobacter lwoffii* (*A. lwoffii*) and *Acinetobacter baumannii* (*A. baumannii*) and of these *E. coli*; *P. mirabilis*, *P. aeruginosa* were most frequently found in various types of clinical solid waste. The Gram positive bacteria detected were *Staphylococcus aureus* (*S. aureus)*, *Streptococcus mutans* (*S. mutans*), *Staphylococcus epidermidis* (*S. epidermidis*), *Enterococcus faecalis* (*E. faecalis*), *Lysinibacillus sphaericus* (*L. sphaericus*), *Streptococcus pyogenes* (*S*. *pyogenes*), and *Streptococcus agalactiae* (*S. agalactiae*).

**Table 1:** Detection of bacteria in clinical solid waste.

No.	Type of clinical solid waste	Microorganisms
1	General waste	*Escherichia coli*
		*Proteus mirabilis*
		*Pseudomonas aeruginosa*
		*Staphylococcus aureus*
2	Dental solid waste	*Streptococcus mutans*
		*Staphylococcus epidermidis*
		*Salmonella* spp.
		*Enterococcus faecalis*
		*Klebsiella pneumoniae*
		*Serratia liquefaciens*
		*Escherichia coli*
		*Staphylococcus aureus*
3	Microbiological lab waste	*Streptococcus pyogenes*
		*Streptococcus mutans*
		*Escherichia coli*
		*Klebsiella pneumoniae*
		*Serratia liquefaciens*
		*Serratia marcescens*
		*Proteus mirabilis*
		*Enterococcus faecalis*
		*Staphylococcus aureus*
		*Staphylococcus epidermidis*
		*Staphylococcus lentus*
		*Pseudomonas aeruginosa*
		*Lysinibacillus sphaericus*
		*Pseudomonas aeruginosa*
		*Acinetobacter lwoffii*
		*Acinetobacter baumannii*
4	Clinical solid waste from ICU unit	*Acinetobacter baumannii*
		*Klebsiella pneumoniae*
		*Proteus mirabilis*
		*Staphylococcus aureus*
		*Pseudomonas aeruginosa*
		*Escherichia coli*
		*Serratia marcescens*
5	Clinical solid waste from Dermatology unit	*Streptococcus pyogenes*
		*Staphylococcus aureus*
		*Staphylococcus epidermidis*
		*Pseudomonas aeruginosa*
6	Clinical solid waste from Isolation wards	*Streptococcus pyogenes*
		*Staphylococcus aureus*
		*Staphylococcus epidermidis*
		*Enterococcus faecalis*
		*Escherichia coli*
		*Klebsiella pneumoniae*
		*Enterococcus faecalis*
		*Proteus mirabilis*
		*Pseudomonas aeruginosa*
		*Lysinibacillus sphaericus*
7	Clinical solid waste from Obstetrical & Gynecology ward	*Escherichia coli*
		*Pseudomonas aeruginosa*
		*Proteus mirabilis*
		*Streptococcus agalactiae*

*S. aureus* was commonly found in waste collected from general solids, Microbiological lab, ICU and Dermatology units and Dental, Isolation, Obstetrical & Gynecology wards. *S. agalactiae* was only detected in clinical waste collected from the Obstetrical & Gynecology ward.

Generation of clinical solid waste in healthcare facilities is increasing. Cautious handling and disposal is not a compromise due to its infectious nature. The survival time life of pathogenic microorganisms present in clinical solid waste has not been fully discussed in the literature. The survival time of pathogenic microorganisms will indicate its presence in clinical solid waste and help determine the safe procedures for handling and disposal of the waste. The present study demonstrated the presence pathogenic bacteria in various types of clinical solid waste. Sixteen different types of bacteria were detected in clinical solid waste. It was observed that many of the bacterial agents were common in the clinical solid wastes studied. The present study also detected the presence of *E. coli*, *P. mirabilis*, *P. aeruginosa* and *S. aureus* in general waste. Contrary to the present findings, previous studies reported that the general waste generated in a healthcare facilities are free from infectious bacterial agents [2,5,7,16,17]. However, Park *et al.* [19] determined the presence of *Pseudomonas* spp., *Lactobacillus* spp., *Staphylococcus* spp., in various types of clinical wastes and the bacterial agents in general waste were similar to the pathological waste. Alagoz and Kocasoy [22], also determined the presence of coliform bacteria, *E. coli*, *Enterobacter*, *Pseudomonas* spp., *S. aureus*, *B. cereus*, *Salmonella* spp. in clinical waste. Vieira *et al.* [23] detected various types bacterial agents in dental waste including *Enterobacter* spp., *Salmonella* spp., *Klebsiella* spp., *Pseudomonas* spp., *Serratia* spp., *Proteus mirabilis*, *Escherichia* spp., *Staphylococcus* spp., *Enterococcus* spp. and *Streptococcus* spp. Earlier researcher has mentioned that among the detected bacteria, *P. aeruginosa*, *Klebsiella pneumoniae*, *P. mirabilis*, *S. aureus*, *Enterococcus faecalis*, *Streptococcus pyogenes*, *Serratia marcescens*, *Acinetobacter baumannii*, *Escherichia coli* can be etiological agents for nosocomial infections [24,25,26,27,28,29,30,31,32,33,34,35]. *Pseudomonas aeruginosa* is an opportunistic and highly adaptive pathogenic bacteria, which is a major hospital acquired pathogen. It is resistant to various environmental stresses, and able to survive in the environment without a host for more than 40 days [24,25]. Other researchers claimed *Klebsiella pneumoniae* as an opportunistic pathogen. It is a multi drug resistant nosocomial organism with the ability to produce extended-spectrum beta-lactamases (ESBL) [26,27]. *P. mirabilis* is the second most common cause of urinary tract infections and one of the important causes of nosocomial infections [28]. Tang *et al.* [29] reported *S. aureus* is capable of causing life-threatening infectious diseases such as pneumonia, meningitis, osteomyelitis, endocarditis, toxic shock syndrome, bacteremia, and sepsis. *Enterococcus faecalis* is a nosocomial opportunistic pathogen which can cause endocarditis and bacteremia, urinary tract infections, meningitis, and other infections in humans. *Streptococcus pyogenes* can produce a wide variety of systemic and cutaneous infections [30,31]. *Serratia marcescens* causes endemic and epidemic nosocomial infections and known to cause infections of the respiratory tract, urinary tract, wounds, and bloodstream [32,33]. *Acinetobacter baumannii* was reported to be a major cause of hospital-acquired infections and may cause a broad range of infections, including pneumonia, lung abscess, meningitis, septicaemia, urinary tract and wound infections [34,35]. One of the Gram positive spore-forming bacterium identified in the waste *Lysinibacillus sphaericus* is reported to be an accidental human pathogen which can causes bactermia, meningitis, pseudotumors, food infection [36].

The bacterial strains detected in clinical sharp waste are presented in Table 2. The entire isolated DNA which was used for 16s rDNA sequencing were analyzed. The A_260_/A_280 _ratio was found to be 1.8 to 2.0, which is within the acceptable value. Results of 16s rDNA sequencing on 11 isolates were found to be ≥99% similar to the sequences listed in the NCBI database. Eight isolates were found to be <99% similar to the sequence listed in the NCBI database. It was observed that of the 19 isolates sequenced, seven strains (36.84%) were *E. coli*, which represents the most abundant type of bacteria isolated from the collected samples. The other bacterial strains were *K. pneumoniae* (21.05%), *L. sphaericus* (10.53%), *P. mirabilis* (10.53%), *S. marcescens* (10.53%) and *S. aureus* (10.53%).

**Table 2:** 16s rDNA base identification of bacteria in clinical sharp waste.

Exhibiting ≥99% 16s rDNA sequence homology with the blast results		Exhibiting <99% 16s rDNA sequence homology with the blast results similarity	
Bacterial Strain	Accession ID	Bacterial Strain	Accession ID
*Escherichia coli* strain D i14	CP002212.1	*Escherichia coli* strain 114	JN180963.1
*Escherichia coli* strain KO11FL	CP002970.1	*Escherichia coli* strain APEC O1	CP000468.1
*Escherichia coli* strain UM146	CP002167.1	*Escherichia coli* strain BW2952	CP001396.1
*Klebsiella pneumoniae* strain AGR/IICT/1	JQ973896.1	*Escherichia coli* strain IHE3034	CP001969.1
*Klebsiella pneumoniae* strain mcp11d	EF419182.1	*Klebsiella pneumoniae* strain ELA-21o	FJ195012.1
*Proteus mirabilis* strain HH134	HQ407314.1	*Klebsiella pneumoniae* strain T89 16S	HQ407264.1
*Proteus mirabilis* strain HH139	HQ407311.1	*Lysinibacillus sphaericus* strain NBIGP 6	JF304288.1
*Serratia marcescens* strain 21-3	JF429937.1	*Lysinibacillus sphaericus* strain SP19_LP11	JQ289050.1
*Serratia marcescens* strain CTC639-K12	JQ917918.1		
*Staphylococcus aureus* strain HO 5096 0412	HE681097.1		
*Staphylococcus aureus* strain NBRC 102140	AB681714.1		

Clinical sharp waste is considered highly hazardous to healthcare workers due to its potential of infection by injury. The present study was conducted to determine the presence of bacterial agents in sharp waste using 16s rDNA sequencing in order to define the specific bacterial strains present. *Escherichia coli*, *Klebsiella pneumoniae*, *Proteus mirabilis*, *Lysinibacillus sphaericus*, *Serratia marcescens*, and *Staphylococcus aureus* were among the nosocomial strains of pathogenic bacteria detected in clinical sharp waste. Many of the detected bacterial strains of *Klebsiella pneumoniae*, *Proteus mirabilis*, *Serratia marcescens*, and *Staphylococcus aureus* are classified as resistant to various environmental stresses and able to produced ESBL [26,28,29,33,37]*.* Park *et al.* [19] reported that viral agents cannot survive in clinical sharp waste without a host organism as opposed to bacterial agents that could multiply and survive for a long time with environmental stress in nutritious clinical waste. The transmission of viral pathogens, including human immunodeficiency virus, hepatitis C virus, and HBV are caused during unsafe handling of the contaminated sharps such as needles and syringes. However, its transmission could be minimized by using the disposable syringes and protective materials to avoid the accidental exposure during handling. In this study the detection of the viral pathogen in clinical solid waste was not conducted.

The most commonly used technology to treat clinical solid waste is incineration. Incineration is unable to inactivate heat resistance pathogenic bacteria, those are released to the environment through stack gas and bottom ash [14,38,39]. It is the common practice worldwide to dispose the general waste along with municipal waste via landfilling or open dumping without consideration of the presence of bacteria. There are some healthcare facilities that recycle the general solid waste materials [40,41]. However, the presence of pathogenic bacteria agent in general waste would be damaging to human health and the environment. Therefore, the present study strongly recommends sterilization of healthcare waste (whether general of clinical solid waste) at its generation source prior to its disposal in order to eliminate nosocomial infections and environmental pollution from clinical and general waste.

## 4. Conclusions

This study confirmed the presence of pathogenic bacteria in various types of clinical solid waste, general waste and clinical sharp waste. Data from the presence of pathogenic bacteria in healthcare waste revealed that clinical solid wastes contain various types of nosocomial and opportunistic pathogenic bacteria. *Pseudomonas aeruginosa*, *Staphylococcus aureus* was also detected in general waste. The findings of the present study recommend that clinical solid waste; general waste and clinical sharp waste be sterilized at the point of generation in order to eliminate nosocomial infections and possible environmental pollution.
